# Current Updates on Choanal Atresia

**DOI:** 10.3389/fped.2015.00052

**Published:** 2015-06-09

**Authors:** Kelvin M. Kwong

**Affiliations:** ^1^Division of Otolaryngology Head and Neck Surgery, Department of Surgery, Rutgers Robert Wood Johnson Medical School, New Brunswick, NJ, USA

**Keywords:** choanal atresia, human, diagnosis, children, management

## Abstract

Choanal atresia (CA) is a relatively uncommon but well-recognized condition characterized by the anatomical closure of the posterior choanae in the nasal cavity. Since the original description back in the early eighteenth century, there have been controversies regarding its exact pathogenesis, the optimal surgical approach, and the use of adjunct treatments such as post-surgical stenting and anti-neoplastic agents, despite of abundant literature available. The emergence and development of new technologies play a significant role in the management of this condition. This review provides a comprehensive clinical update on CA and identifies areas for future study based on the existing available literature.

## Introduction

Choanal atresia (CA) is a well-recognized etiology for congenital nasal airway abnormality that could have various clinical presentations ranging from acute airway obstruction to chronic recurrent sinusitis. This disease entity was first described by Roederer in 1755 (1) while Oto et al. further characterized the anomaly in relation to the deformity of the palatine bones in 1829 (2). Emmert et al. in 1851 was the first to demonstrate the use of curved trochar in tranasal repair of bilateral CA in a 7-year-old boy (3). By 1920s, most of CA clinical features were recognized, and four surgical techniques including trans-nasal, trans-septal, trans-palatal, and trans-maxillary approach had been devised (4). Since the first description back in eighteenth Century, there has been abundance of literature describing various aspects of CA. However, there are still controversies on the exact pathogenesis, the effectiveness on various surgical techniques, and the use of post-surgical stenting and anti-neoplastic agent as adjunct for the surgical treatments. The purpose of this review is to provide a comprehensive clinical update on CA and to identify areas for future study based on the existing literature available currently.

## Relevant Anatomy and Epidemiology

Choanal atresia, defined as the anatomical closure of the posterior choanae in the nasal cavity, is relatively uncommon disease entity with an estimated incidence of 1:5000–7000 birth (5). Historically reported in 1910, the deformity was made up of 90% bony and 10% membranous atresia (6). With a modern workup of computer tomography, a retrospective study reviewing the CT and histologic specimens in 63 patients showed a 29% pure bone atresia, 71% mixed membranous and bone atresia with no pure membranous atresia present (7). Anatomic boundaries of the posterior choanae include the undersurface of the body of the sphenoid bones superiorly, the medial pterygoid lamina laterally, the vomer medially, and the horizontal portion of the palatal bone inferiorly. The actual narrowing could be caused by one of the abovementioned bony components. Correctly identifying and addressing the point of obstruction is the key to surgical success. Interestingly, the characteristics of CA follow a “2–1” rule, namely the ratio of unilateral to bilateral CA, female to male and the right sided to the left sided CA (8). In a study of 129 patients with choanal atresia and stenosis (CA/S), about 51% of studied patients had another congenital anomalies, the most common being Coloboma, Heart disease, Choanal atresia, Mental and growth retardation, Genital hypoplasia, Ear deformities (CHARGE) syndrome (9). Bilateral CA/S was more commonly seen in patients in whom other congenital anomalies are identified while unilateral CA/S occurred more frequently in isolated cases (9).

## Embryology and Pathogenesis

Development of the nasal cavity starts with neural crest cells migration from their origin in the dorsal neural folds at about 3.5 weeks of fetal life. During the subsequent 2 weeks, nasal processes or placodes invaginate to form the nasal pits. The nasal pits burrow deeper within the mesenchyme while nasobuccal membrane normally will rupture to create a nasal cavity with the primitive choana (10). Four basic theories have been accepted over the years (8): (1) Persistence of the buccopharyngeal membrane from the foregut. (2) Abnormal persistence or location of mesoderm forming adhesions in the nasochoanal region. (3) Abnormal persistence of the nasobuccal membrane of Hochstetter. (4) Misdirection of neural crest cell migration. Various molecular or genetic models have been studied to give further insights in the pathogenesis of CA.

### The role of retinoic acid

Retinoic acid deficiency during gestation period has been known to induce numerous malformations (11). Retinoic acid (RA) produced from vitamin A by retinaldehyde dehydrogenase (Raldh) is important for ontogenesis and homeostasis of numerous tissues (12). Using a mouse model, Dupe et al. demonstrated that Raldh3 knockout suppressed RA synthesis and caused CA due to persistence of nasal fins, whose rupture normally allowed the communication between nasal and oral cavities (12). Dupe et al. proposed that the impaired RA synthesis caused over-expression of fibroblast growth factor 8 (FGF-8), which in turned led to persistent nasal fins. They also demonstrated that the CA in the Raldh3 knockout mice could be prevented by maternal treatment of RA (12). The role of FGF-8 in CA development was further supported by the observation of prevalent CA among patients with craniosynostosis syndromes as a result of the elevated level of FGF-8 expression (13).

### Thioamides and CA

Thioamides, such as methimazole, carbimazole, and propylthiouracil, are commonly used as medical treatment for hyperthyroidism. Their potential association with CA was described by several case reports of CA in the newborns from mothers who had prenatal use of thioamides (14–16). In a more recent case-control study, Barbero et al. found prenatal exposure to maternal hyperthyroidism treated with methimazole was associated with CA development (17). However, based on their studied cases and a critical literature review, it was proposed that the mother’s hyperthyroidism rather than the methimazole treatment might be the causal factor for CA (17). Elevated thyroid-stimulating hormones (TSH) level was associated with increased level of FGF, FGF receptor, and other proliferating growth factors, which hypothetically form the basis for CA development (18).

However, further studies are required to further delineate the causes and pathogenesis of CA.

## Clinical Presentation and Diagnosis

Clinical presentation of CA varies from acute airway obstruction to chronic recurrent sinusitis depending on whether CA is unilateral, bilateral, or associated with other coexisting airway abnormalities, as often seen in patients with CHARGE syndrome and craniofacial anomalies.

### Bilateral CA

**Figure 1:**
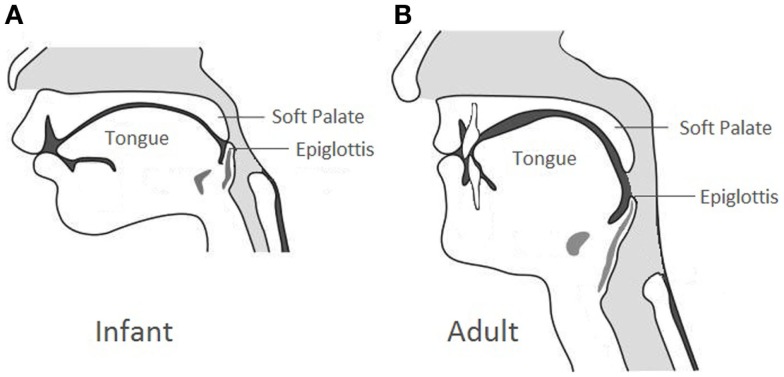
**Anatomical comparison between the infant larynx and the adult larynx**.

Due to the elevated laryngeal position compared to the adult counterpart (Figure 1), newborns are obligate nose breathers until mouth breathing is established with the descent of the larynx approximately 4–6 weeks of life. In case of bilateral CA, infants can have acute respiratory distress with intermittent cyanosis characteristically relieved by crying. Feeding difficulty can be the initial alerting event in which the infants can present with progressive airway obstruction and choking during feeding because of their inability to breathe and feed simultaneously. Neonates with bilateral CA can also present with a history of multiple failed extubation attempts, especially in those with secondary airway issues.

### Unilateral CA

Infants with unilateral CA, rarely present with neonate respiratory distress, are often diagnosed later on in life. The most common presentation is chronic unilateral nasal obstruction, persistent mucoid rhinorrhea, and/or a history of chronic sinusitis. Occasionally, the correct diagnosis could not be reached until adulthood due to the non-specific symptoms of unilateral nasal obstruction. Given the relatively low morbidity of unilateral CA, its definitive treatment is usually delayed until later age as the chance of successful surgical repair increases with age (i.e., more favorable anatomy later on in life) (19).

### CA with multiple congenital anomalies

Burrow et al. studied 129 patients with CA/S and demonstrated that multiple congenital anomalies are commonly found among these patients, especially in those with bilateral CA (9). Within this particular group of patients, about 34% had other airway abnormalities, such as tracheomalacia, laryngomalacia, and subglottic stenosis and about 21% had craniofacial abnormalities including CHARGE, Treacher Collins, Pfeiffer, Apert, Mandibulofacial dysostosis, and Crouzon syndromes (9). Given the complex airway abnormality, this subgroup of patients always presents with more acute and severe respiratory symptoms and requires stable alternative airway management such as tracheostomy in addition to the surgical correction of CA.

### Diagnosis

Initial clinical evaluation includes introduction of a six or eight Fr suction catheter via the nostrils, methylene blue dye test, cotton wisp test, and laryngeal mirror test. The distance of encountered resistance can provide insights into the etiology of nasal obstruction. An obstruction at approximately 1–2 cm from the ala rim in neonates is most likely deflection of the nasal septum or inferior turbinate while 3–3.5 cm from the alar rim indicates obstruction at the level posterior choanae. Flexible nasal endoscopy in a patient with proper preparation, such as nasal decongestion and mucous suctioning, allows direct visualization of the point of obstruction in the nasal passage and confirms the presence of an atretic plate in the choana. Therefore, it has become the preferred method for making the diagnosis.

**Figure 2:**
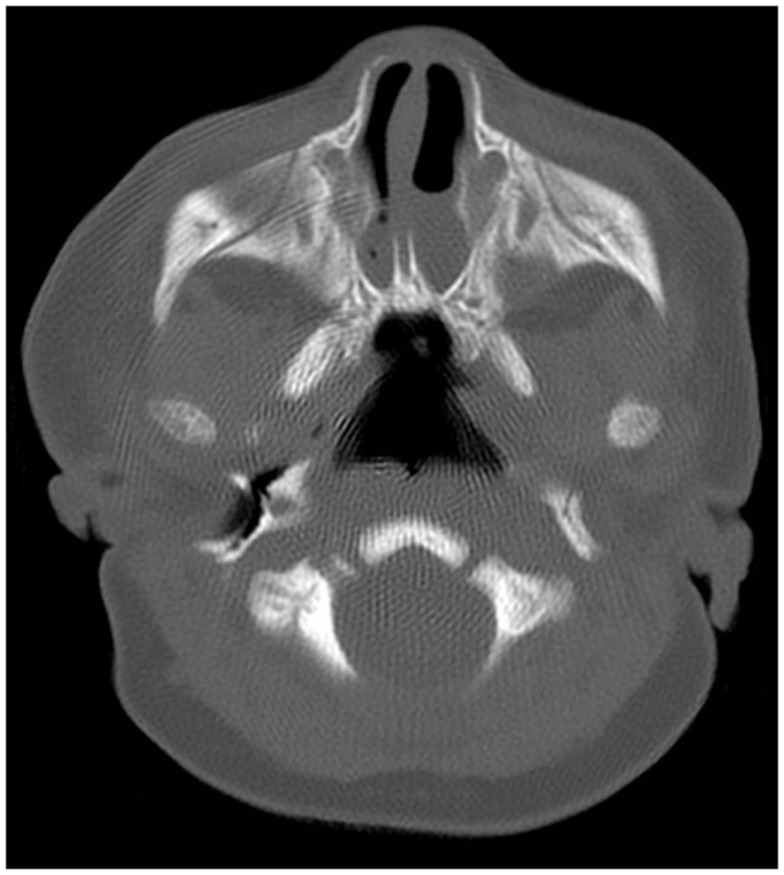
**Axial CT scan of the nasal cavity in bone window at the level of choana**.

Definitive evaluation is achieved with a CT of sinuses utilizing 2–5 mm cuts in patients with proper nasal preparation. CT demonstrated CA in form of the thickening of the anterior portion of the pterygoid plates and the enlargement of the posterior portion of the vomer, with or without membranous involvement (Figure 2). To obtain a more precise anatomic definition of CA, Slovis et al. reviewed and compared CT findings between 11 CA patients and 66 control patients (20). The mean choanal airspace of newborn, measured between lateral nasal wall and the vomer, was 0.67 mm, which increased 0.27 mm per year up to 20 years old. The mean vomer thickness was 2.3 mm in children with age <8 years and 2.8 mm in children ≥8 years. In patients with CA, choanal airspace was absent in bony atresia and 1/3 of the norm in membranous atresia while the mean vomer thickness was 6.0 mm in bony atresia and 3.0 mm in membranous counterpart (20). A bony atretic plate may vary from 1 to 12 mm depending on the bony changes of the medial pterygoid lamina and vomer (20).

Besides delineating the nature and severity of CA, CT is also useful in differentiating other causes of nasal obstruction from CA. Differential diagnoses include pyriform aperture stenosis, nasolacrimal duct cysts, turbinate hypertrophy, septal dislocation and deviation, antrochoanal polyp, or nasal neoplasm.

## Management

### Initial airway management

Infants with bilateral CA can present as an airway emergency at birth. The goal of initial treatment for these patients is to maintain an adequate airway via the oral route. The preferred oral airway is McGovern nipple, an intraoral nipple with a large opening by cutting its end off, secured in the mouth with ties around the infant’s occiput. A small feeding tube can be placed through another hole in the nipple or alongside the nipple to provide feeding needs. If patient fails to maintain an adequate airway with McGovern nipple, endotracheal intubation should be considered as the initial management.

Tracheostomy may be required if the definitive surgical treatment needs to be deferred due to patient’s other comorbidities such as cardiopulmonary instability and multi-level airway obstruction. Patients with bilateral CA and CHARGE syndrome are more likely to fail atresia repair because of their (i) more contracted nasopharynx in a lateral and/or vertical dimension, (ii) narrower posterior choanal region than those with isolated CA and (iii) poor tongue/pharyngeal muscle control (21). Asher et al. in a retrospective review of 16 patients with CHARGE syndrome demonstrated that early repair of their CA was rarely successful. These patients had a propensity for airway instability leading to hypoxic events. Authors in the study therefore recommended early tracheotomy rather than early CA repair to secure the airway for CA patients with CHARGE syndrome (22).

### Surgical management

Since the first attempt of CA surgical repair in mid-nineteenth century, there have been hundreds of articles published on the outcomes and effectiveness of various surgical approaches. However, there is no consensus on the recommended surgical techniques because of the several limitations in the existing literature. Majority of studies were single surgeon or institution series with small sample size. Different surgical techniques, variable duration of stenting or use of adjunct therapy (e.g., mitomycin) and lack of standardized outcome measures (i.e., definition for choanal patency and surgical failure) make comparison and meaningful interpretation of various studies difficult, and sometimes impossible. A recent Cochrane review provided a similar conclusion regarding the above limitations of the existing literature on CA surgery and recommended a unified effort in multicenter randomized controlled trials that test the effectiveness and safety of different surgical techniques in patients with CA (23).

### Transnasal puncture

**Figure 3:**
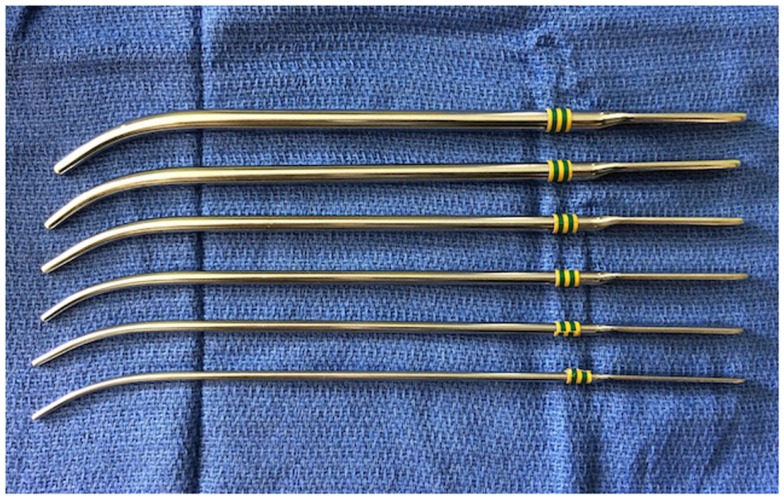
**Urethral sounds in various sizes**.

First, CA repair was performed using a simple puncture transnasally by Emmert in 1851 (3). Urethral sounds (Figure 3) or Fearson dilators are used to puncture the atretic plate blindly via the nostrils. This approach has been used reliably in neonates with bilateral CA (24). Over time, the technique has been refined with the use of 120° endoscope or laryngeal mirror to visualize the nasopharyngeal side of the atretic plate to ensure the safe passage of the dilators. Post-op stenting or combination with endoscopic resection is recommended because of the high restenosis rate. This approach is useful in patients with thin membranous and bony CA especially in very small infants where direct visualization with endoscope could be difficult. However, this approach is not recommended for thick bony atretic plate or patients with CHARGE syndrome as Hengerer et al. reported in their series showing that 100% (seven out of seven) of the CHARGE patients had restenosis (8). Potential complications of transnasal puncture include septal and/or turbinate injury, intranasal adhesion, clival injury causing Gradenigo syndrome, CSF leakage, and meningitis from penetration and fracture of the perpendicular plate and/or cribriform plate of ethmoids (24).

### Transpalatal repair

Transpalatal CA repair was first described by Owens in 1965 as an approach to optimize the exposure of the atretic plate for repair. A U-shaped mucosal flap posteriorly based on greater palatine vessels is raised beyond the hard and soft palate junction. The palatine bones posterior to the greater palatine foramina, the atresia plates, the posterior vomer and the medial pterygoid plates are carefully drilled using a diamond burr. Despite of the high reported primary success rate up to 84% (25), the potential complications of this approach could be significant. Palatal incisions may have a banding effect on maxillary growth causing cross-bite and high palatal arch deformity (8). Freng et al. showed that cross bite frequency in 55 CA patients (52%) treated by transpalatal repair was significantly higher than that of the 265 controls (4%) (26). Other potential complications also include palatal flap breakdown, fistula, palatal muscle dysfunction, and velopharyngeal insufficiency (27). Due to these morbid complications, this surgical approach is not recommended for children younger than 6 years (4, 10, 26) Advancement of miniature endoscope and powered instrument allows the development of transnasal endoscopic approach for CA repair.

### Transnasal endoscopic repair

**Figure 4:**
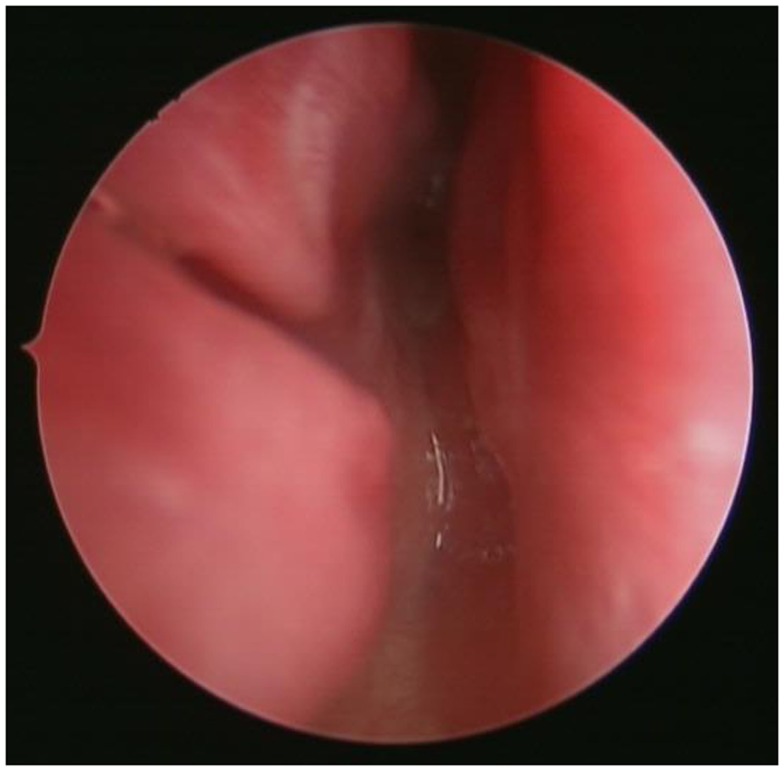
**Endoscopic view of the atretic plate of the choanal atresia**.

Park et al. conducted a survey of pediatric otolaryngologists belonging to the American Society of Pediatric Otolaryngology and demonstrated that endoscopic techniques and the use of powered instrumentation have become the primary procedure by most surgeons (25). The use of endoscopic techniques for transnasal CA repair was first demonstrated by Stankiewicz (28). Since then, there have been numerous reports on different modifications of the endoscopic techniques. After adequate nasal decongestion, a 2.9 or 4.0 mm Hopkins rigid endoscope is introduced in the nostril to visualize the atretic plate (Figure 4). A laterally based mucosal flap is then raised to expose the bony part of the atretic plate. The thinnest section of the atresia, usually found at the junction of the hard palate and vomer below the tail of middle turbinate, is the ideal point of entry into the nasopharynx. Posterior bony septum (i.e., vomer) was removed to create a “neo-unichoana” (29) using powered instrument. No more than 1/3 of the bony septum is removed to prevent the potential adverse effect on the nasal growth centers. Avoiding bone ridges and covering the exposed bone surface with mucosa are essential to prevent post-operative restenosis (30).

In case the nostril is too small to accommodate both endoscope and microdrill ipsilaterally, a posterior septal window can be created by using a sickle knife or cottle dissector (31). Then, the posterior bony septum was enlarged by using either microdrill or backbiting forcep in one nostril under direct visualization using the endoscope in the other nostril. Once optimal exposure of the atretic plate is created, the rest of repair proceeds with the creation of a “neo-unichoana” in the similar fashion.

Various studies have reported primary repair success rates ranging from 67 to 88% (32). Mean success rate with transnasal endoscopic repair was 85.3% in a total of 238 cases in a meta-analysis of 20 studies (33). The primary repair success rate is difficult to interpret and compared across various studies because there is no standard definition of “surgical failure.” Some authors consider revision as removal of excessive granulation while others define it as any procedure requiring general anesthesia including stent replacement or removal (34). Post-operative restenosis remains a common complication of the endoscopic CA repair (35, 36). Risk factors for restenosis include nasopharyngeal reflux, gastroesophageal reflux, age <10 days (associated with limited visualization in noses of neonates and limited resection of the vomer), bilateral CA with purely bony atretic plate, the presence of associated malformations (19, 36–38). Frequent post-operative use of nasal saline irrigation and periodic endoscopic surveillance or second look procedures can improve the primary repair success and reduce the rate of restenosis (30).

### Post-operative stenting

The use of post-operative stent in CA repair is a controversial topic in the existing literature. Its use has traditionally been one of post-surgical adjunct measures to reduce the chance of restenosis. In Park et al.’s survey of 95 pediatric otolaryngologists in 2000, 92 of them routinely used stents to maintain the choanal patency after CA repair. However, there is also disagreement in the stenting duration, the type of stents used and even the techniques in which stents are secured in the nostrils (38). These inconsistencies may underline the reason why there has been no clear-cut evidence on the effectiveness of using stents after CA repair (5). Indeed, data from the recent studies suggest that there is no difference in restenosis rates regardless of stenting (33, 34, 39). Bedwell et al. in his review demonstrated that outcomes were good regardless of whether post-operative stents are used (39). Repair without stenting reduces the intensity of post-operative management and avoids the potential for stent-related complications, such as discomfort, localized infection and ulceration, circumferential scar or granulation tissue formation (40). A meta-analysis of 238 cases from 20 studies by Durmaz et al. failed to show the use of post-operative stents provided any significant difference in the surgical outcome of CA repair (33). A combination of close post-operative follow-up, revision endoscopy to remove nasal crusting 1 week after the primary repair, and frequent nasal saline irrigation was the key to successful management of CA without stenting (36).

### Mitomycin C

Isolated from Streptomyces bacterial species, mitomycin C is an aminoglycoside, which inhibits DNA synthesis by generating oxygen radicals that alkylate and crosslink DNA. It has been shown to inhibit fibroblast proliferation and migration at the cellular level. Topical application of mitomycin C is used clinically in reducing post-surgical glottis and subglottic stenosis and also preventing recurrent respiratory papillomatosis. Studies in early 2000s showed improved surgical outcome of CA repair with mitomycin C use (41, 42). However, none of those studies had a proper control group to demonstrate the improved surgical outcome was caused by the use of mitomycin C. Subsequent studies failed to show significant difference in restenosis rate between the mitomycin group and the control group (43–45). Similarly, Carter et al. in their recent study had the same conclusion about restenosis rate while showing decreased granulation tissue formation and fewer subsequent surgical debridements with topical mitomycin C treatment (45). The long-term effect of this potentially carcinogenic medication as the treatment of a benign condition in children remains unclear (46). Currently, the routine use of mitomycin C as an adjunct for surgical CA repair is not supported by the available evidence.

### Surgery with CT-image guidance

Navigation with CT-image has been commonly used in endoscopic sinus and anterior skull base surgeries in otolaryngology. There are case reports of successful use of CT-image guidance during endoscopic transnasal CA repair in selected patients with special anatomic considerations, such as low-birth weight neonates and infants with Treacher Collins Syndrome (47, 48). Most of the CA cases can be done safely with high quality pre-operative CT scans and good visualization using appropriately sized endoscope and instruments (5).

### Laser-assisted surgery

Laser has been used in endonasal surgery more than 30 years. Healy et al. first described and demonstrated the feasibility of using CO_2_ laser in management of CA back in 1978 (49). However, bone ablation was not possible when bony atretic plate was >1 mm in thickness. Other types of laser, such as Nd-YAG, holmium-YAG, potassium titanyl phosphate (KTP), and contact diode laser (CDL) for repair of CA have been described (35, 50–52). The advancement of miniature endoscope and small fiberoptic delivery system including CO_2_ laser allows good visualization and precise delivery of the laser with improved maneuverability. However, the lack of significant advantage over the conventional powered instruments, increased fire risks, and reported deaths from air embolism related to Nd-YAG laser use limit the wide acceptance of laser-assisted CA repair (5, 53). As the technology continues to evolve and future research provides more outcome data, lasers may play a further role in the CA repair.

## Summary

Choanal atresia is a well-recognized etiology for congenital nasal obstruction. Since the original description back in the early eighteenth century, there have been controversies regarding its exact pathogenesis and the optimal surgical management in spite of abundant literature available. Despite of the increased molecular understanding of CA formation in recent years, further research in the genetic component of pathogenesis may help to elucidate the disease process more clearly. Moreover, the lack of randomized controlled trials makes it difficult to demonstrate the superiority of a specific surgical approach for CA repair. Current trends in treatment suggested a predilection toward the transnasal endoscopic approach with the use of miniature endoscope and micro-powered instruments. Creation of neo-unichoana with the use of mucosal flap, frequent post-operative use of nasal saline irrigation and periodic endoscopic surveillance or second look procedures can improve the primary repair success. The use of post-operative stents is decreasing in light of the evidence on the increased stent-related complications, such as local infection, synechiae, and granulation tissue formation. The effectiveness of mitomycin C has not been adequately proven based on the current literature. As the technology is becoming more sophisticated, CT-guided and laser-assisted CA surgery may play a further role especially in patients with challenging anatomy, such as low-birth weight preterm infants and neonates with additional craniofacial anomaly.

## Conflict of Interest Statement

The author declares that the research was conducted in the absence of any commercial or financial relationships that could be construed as a potential conflict of interest.

## References

[1] Flake CG Ferguson CF Congenital choanal atresia in infants and children. Ann Otol Rhinol Laryngol (1964) 73:458–73. 10.1177/000348946407300216 14196687

[2] Otto A Lehrbach D Pathologischen Anatomic des Menschen und der Thiere. (Vol. 1). Berlin: Recker (1830). p. 181–3.

[3] Emmert C Stenochorie und Atresie der Choannen, Lehrbach der Speciellen Chirurgie. (Vol. 2). Stuttgart: Dann (1854). p. 535–8.

[4] Pirsig W. Surgery of choanal atresia in infants and children: historical notes and updated review. Int J Pediatr Otorhinolaryngol (1986) 11(2):153–70. 10.1016/S0165-5876(86)80010-6 3744697

[5] Ramsden JD Campisi P Forte V Choanal atresia and choanal stenosis. Otolaryngol Clin North Am (2009) 42(2):339–52,x. 19328897 10.1016/j.otc.2009.01.001

[6] Fraser J Congenital atresia of the choanae. Br Med J (1910) 2:1968–71.20765374

[7] Brown OE Pownell P Manning SC. Choanal atresia: a new anatomic classification and clinical management applications. Laryngoscope (1996) 106(1 Pt 1):97–101. 10.1097/00005537-199601000-00019 8544637

[8] Hengerer AS Brickman TM Jeyakumar A. Choanal atresia: embryologic analysis and evolution of treatment, a 30-year experience. Laryngoscope (2008) 118(5):862–6. 10.1097/MLG.0b013e3181639b91 18197129

[9] Burrow TA Saal HM de Alarcon A Martin LJ Cotton RT Hopkin RJ. Characterization of congenital anomalies in individuals with choanal atresia. Arch Otolaryngol Head Neck Surg (2009) 135(6):543–7. 10.1001/archoto.2009.53 19528400

[10] Hengerer AS Strome M Choanal atresia: a new embryologic theory and its influence on surgical management. Laryngoscope (1982) 92(8 Pt 1):913–21. 10.1288/00005537-198208000-00012 7098739

[11] Wilson JG Roth CB Warkany J An analysis of the syndrome of malformations induced by maternal vitamin A deficiency. Effects of restoration of vitamin A at various times during gestation. Am J Anat (1953) 92(2):189–217. 10.1002/aja.1000920202 13030424

[12] Dupe V Matt N Garnier JM Chambon P Mark M Ghyselinck NB. A newborn lethal defect due to inactivation of retinaldehyde dehydrogenase type 3 is prevented by maternal retinoic acid treatment. Proc Natl Acad Sci U S A (2003) 100(24):14036–41. 10.1073/pnas.2336223100 14623956 PMC283541

[13] Hehr U Muenke M Craniosynostosis syndromes: from genes to premature fusion of skull bones. Mol Genet Metab (1999) 68(2):139–51. 10.1006/mgme.1999.2915 10527665

[14] Greenberg F. Choanal atresia and athelia: methimazole teratogenicity or a new syndrome? Am J Med Genet (1987) 28(4):931–4. 10.1002/ajmg.1320280419 3688031

[15] Chabrolle JP Bruel H El Khoury E Poinsot J Amusini P Benouada A [Methimazole and choanal atresia]. Arch Pediatr (2003) 10(5):463–4. 10.1016/S0929-693X(03)00097-6 12878343

[16] Wilson LC Kerr BA Wilkinson R Fossard C Donnai D. Choanal atresia and hypothelia following methimazole exposure in utero: a second report. Am J Med Genet (1998) 75(2):220–2. 10.1002/(SICI)1096-8628(19980113)75:2<220::AID-AJMG21>3.0.CO;2-Q 9450891

[17] Barbero P Valdez R Rodriguez H Tiscornia C Mansilla E Allons A Choanal atresia associated with maternal hyperthyroidism treated with methimazole: a case-control study. Am J Med Genet A (2008) 146A(18):2390–5. 10.1002/ajmg.a.32497 18698631

[18] Cocks HC Thompson S Turner FE Logan A Franklyn JA Watkinson JC Role and regulation of the fibroblast growth factor axis in human thyroid follicular cells. Am J Physiol Endocrinol Metab (2003) 285(3):E460–9. 10.1152/ajpendo.00519.2002 12746216

[19] Newman JR Harmon P Shirley WP Hill JS Woolley AL Wiatrak BJ. Operative management of choanal atresia: a 15-year experience. JAMA Otolaryngol Head Neck Surg (2013) 139(1):71–5. 10.1001/jamaoto.2013.1111 23329094

[20] Slovis TL Renfro B Watts FB Kuhns LR Belenky W Spoylar J. Choanal atresia: precise CT evaluation. Radiology (1985) 155(2):345–8. 10.1148/radiology.155.2.3983384 3983384

[21] Coniglio JU Manzione JV Hengerer AS. Anatomic findings and management of choanal atresia and the CHARGE association. Ann Otol Rhinol Laryngol (1988) 97(5 Pt 1):448–53. 10.1177/000348948809700503 3178097

[22] Asher BF McGill TJ Kaplan L Friedman EM Healy GB. Airway complications in CHARGE association. Arch Otolaryngol Head Neck Surg (1990) 116(5):594–5. 10.1001/archotol.1990.01870050094014 1691649

[23] Cedin AC Atallah AN Andriolo RB Cruz OL Pignatari SN. Surgery for congenital choanal atresia. Cochrane Database Syst Rev (2012) 2:CD008993. 10.1002/14651858.CD008993.pub2 22336856 PMC12107687

[24] Gujrathi CS Daniel SJ James AL Forte V. Management of bilateral choanal atresia in the neonate: an institutional review. Int J Pediatr Otorhinolaryngol (2004) 68(4):399–407. 10.1016/j.ijporl.2003.10.006 15013604

[25] Park AH Brockenbrough J Stankiewicz J. Endoscopic versus traditional approaches to choanal atresia. Otolaryngol Clin North Am (2000) 33(1):77–90. 10.1016/S0030-6665(05)70208-5 10637345

[26] Freng A. Growth in width of the dental arches after partial extirpation of the mid-palatal suture in man. Scand J Plast Reconstr Surg (1978) 12(3):267–72. 10.3109/02844317809013003 741216

[27] Stieve M Kempf HG Lenarz T. Management of choanal atresia in cases of craniofacial malformation. J Maxillofac Oral Surg (2009) 8(1):52–4. 10.1007/s12663-009-0013-z 23139471 PMC3454022

[28] Stankiewicz JA The endoscopic repair of choanal atresia. Otolaryngol Head Neck Surg (1990) 103(6):931–7. 2126127 10.1177/019459989010300608

[29] Ibrahim AA Magdy EA Hassab MH. Endoscopic choanoplasty without stenting for congenital choanal atresia repair. Int J Pediatr Otorhinolaryngol (2010) 74(2):144–50. 10.1016/j.ijporl.2009.10.027 19945755

[30] Rodriguez H Cuestas G Passali D. A 20-year experience in microsurgical treatment of choanal atresia. Acta Otorrinolaringol Esp (2014) 65(2):85–92. 10.1016/j.otorri.2013.09.005 24556158

[31] El-Ahl MA El-Anwar MW. Stentless endoscopic transnasal repair of bilateral choanal atresia starting with resection of vomer. Int J Pediatr Otorhinolaryngol (2012) 76(7):1002–6. 10.1016/j.ijporl.2012.03.019 22542285

[32] De Freitas RP Berkowitz RG. Bilateral choanal atresia repair in neonates – a single surgeon experience. Int J Pediatr Otorhinolaryngol (2012) 76(6):873–8. 10.1016/j.ijporl.2012.02.063 22444734

[33] Durmaz A Tosun F Yldrm N Sahan M Kvrakdal C Gerek M. Transnasal endoscopic repair of choanal atresia: results of 13 cases and meta-analysis. J Craniofac Surg (2008) 19(5):1270–4. 10.1097/SCS.0b013e3181843564 18812850

[34] Velegrakis S Mantsopoulos K Iro H Zenk J. Long-term outcomes of endonasal surgery for choanal atresia: 28 years experience in an academic medical centre. Eur Arch Otorhinolaryngol (2013) 270(1):113–6. 10.1007/s00405-012-1982-y 22392520

[35] Asma A Roslenda AR Suraya A Saraiza AB Aini AA. Management of congenital choanal atresia (CCA) after multiple failures: a case report. Med J Malaysia (2013) 68(1):76–8. 23466775

[36] Teissier N Kaguelidou F Couloigner V Francois M Van Den Abbeele T. Predictive factors for success after transnasal endoscopic treatment of choanal atresia. Arch Otolaryngol Head Neck Surg (2008) 134(1):57–61. 10.1001/archoto.2007.20 18209138

[37] Kim H Park JH Chung H Han DH Kim DY Lee CH Clinical features and surgical outcomes of congenital choanal atresia: factors influencing success from 20-year review in an institute. Am J Otolaryngol (2012) 33(3):308–12. 10.1016/j.amjoto.2011.08.010 21925765

[38] Corrales CE Koltai PJ. Choanal atresia: current concepts and controversies. Curr Opin Otolaryngol Head Neck Surg (2009) 17(6):466–70. 10.1097/MOO.0b013e328332a4ce 19779346

[39] Bedwell JR Choi SS Are stents necessary after choanal atresia repair? Laryngoscope (2012) 122(11):2365–6. 10.1002/lary.23381 23108881

[40] Schoem SR. Transnasal endoscopic repair of choanal atresia: why stent? Otolaryngol Head Neck Surg (2004) 131(4):362–6. 10.1016/j.otohns.2004.03.036 15467600

[41] Holland BW McGuirt WF Jr. Surgical management of choanal atresia: improved outcome using mitomycin. Arch Otolaryngol Head Neck Surg (2001) 127(11):1375–80. 10.1001/archotol.127.11.1375 11701078

[42] Prasad M Ward RF April MM Bent JP Froehlich P. Topical mitomycin as an adjunct to choanal atresia repair. Arch Otolaryngol Head Neck Surg (2002) 128(4):398–400. 10.1001/archotol.128.4.398 11926914

[43] Kubba H Bennett A Bailey CM. An update on choanal atresia surgery at Great Ormond Street Hospital for Children: preliminary results with mitomycin C and the KTP laser. Int J Pediatr Otorhinolaryngol (2004) 68(7):939–45. 10.1016/j.ijporl.2004.02.010 15183586

[44] Uzomefuna V Glynn F Al-Omari B Hone S Russell J. Transnasal endoscopic repair of choanal atresia in a tertiary care centre: a review of outcomes. Int J Pediatr Otorhinolaryngol (2012) 76(5):613–7. 10.1016/j.ijporl.2012.01.033 22418073

[45] Carter JM Lawlor C Guarisco JL. The efficacy of mitomycin and stenting in choanal atresia repair: a 20 year experience. Int J Pediatr Otorhinolaryngol (2014) 78(2):307–11. 10.1016/j.ijporl.2013.11.031 24367937

[46] Agrawal N Morrison GA. Laryngeal cancer after topical mitomycin C application. J Laryngol Otol (2006) 120(12):1075–6. 10.1017/S0022215106003008 17040596

[47] Westendorff C Dammann F Reinert S Hoffmann J. Computer-aided surgical treatment of bilateral choanal atresia. J Craniofac Surg (2007) 18(3):654–60. 10.1097/scs.0b013e318033856e 17538334

[48] Shah UK Daniero JJ Clary MS Depietro JJ Johnston DR. Low birth weight neonatal choanal atresia repair using image guidance. Int J Pediatr Otorhinolaryngol (2011) 75(10):1337–40. 10.1016/j.ijporl.2011.07.011 21839525

[49] Healy GB McGill T Jako GJ Strong MS Vaughan CW. Management of choanal atresia with the carbon dioxide laser. Ann Otol Rhinol Laryngol (1978) 87(5 Pt 1):658–62. 10.1177/000348947808700510 718063

[50] Fong M Clarke K Cron C. Clinical applications of the holmium: YAG laser in disorders of the paediatric airway. J Otolaryngol (1999) 28(6):337–43. 10604163

[51] Lapointe A Giguère CM Forest VI Quintal MC. Treatment of bilateral choanal atresia in the premature infant. Int J Pediatr Otorhinolaryngol (2008) 72(5):715–8. 10.1016/j.ijporl.2008.01.027 18339432

[52] D’Eredità R Lens MB. Contact-diode laser repair of bony choanal atresia: a preliminary report. Int J Pediatr Otorhinolaryngol (2008) 72(5):625–8. 10.1016/j.ijporl.2008.01.011 18304657

[53] Yuan HB Poon KS Chan KH Lee TY Lin CY. Fatal gas embolism as a complication of Nd-YAG laser surgery during treatment of bilateral choanal stenosis. Int J Pediatr Otorhinolaryngol (1993) 27(2):193–9. 10.1016/0165-5876(93)90136-Q 8258488

